# Anti-coccidial properties and mechanisms of an edible herb, *Bidens pilosa*, and its active compounds for coccidiosis

**DOI:** 10.1038/s41598-019-39194-2

**Published:** 2019-02-27

**Authors:** Wen-Chin Yang, Cheng-Ying Yang, Yu-Chuan Liang, Chu-Wen Yang, Wei-Qun Li, Chih-Yao Chung, Meng-Ting Yang, Tien-Fen Kuo, Chuen-Fu Lin, Chih-Lung Liang, Cicero Lee-Tian Chang

**Affiliations:** 1 Agricultural Biotechnology Research Center, Academia Sinica, Taipei City, Taiwan; 2 Department of Life Sciences, National Chung Hsing University, Taichung City, Taiwan; 3 Department of Veterinary Medicine, College of Veterinary Medicine, National Chung Hsing University, Taichung City, Taiwan; 4 Department of Microbiology, Soochow University, Taipei City, Taiwan; 5 Molecular and Biological Agricultural Sciences, Taiwan International Graduate Program, Academia Sinica, Taiwan, and National Chung-Hsing University, Taichung City, Taiwan; 6 Graduate Institute of Biotechnology, National Chung-Hsing University, Taichung City, Taiwan; 7 Department of Veterinary Medicine, National Chiayi University, Chiayi City, Taiwan; 8 School of Medicine, Chung Shan Medical University, Taichung City, Taiwan

## Abstract

Avian coccidiosis is an economically important disease in the poultry industry. In view of the disadvantages of anti-coccidial drugs in chickens, edible plants and their compounds are re-emerging as an alternative strategy to combat this disease. A previous publication reported that the edible plant *B*. *pilosa* showed promise for use against coccidiosis. Here, we first investigated into the anti-coccidial effects of *B*. *pilosa*. We found that *B*. *pilosa* at 100 ppm or more significantly suppressed *E*. *tenella* as evidenced by reduction in mortality rate, oocyst excretion and gut pathological severity in chickens and its minimum prophylactic duration was 3 days. Next, we explored the mode of action of anti-coccidial mechanism of *B*. *pilosa*. The *E*. *tenella* oocysts were not directly killed by *B*. *pilosa*; however, administration of the plant suppressed oocyst sporulation, sporozoite invasion, and schizonts in the life cycle of *E*. *tenella*. Besides, *B*. *pilosa* boosted T cell-mediated immunity. Finally, we characterized the related anti-coccidial phytochemicals and their mode of action. One of three potent polyynes present in *B*. *pilsoa*, Compound 1 (cytopiloyne), acted against coccidiosis in chickens in a similar manner to *B*. *pilosa*. These data illustrate the anti-coccidial potency and mechanism of *B*. *pilosa* and one of its active compounds, and provide a cornerstone for development of novel herbal remedies for avian coccidiosis.

## Introduction

It is estimated that 50 billion chickens are raised annually worldwide. The parasitic disease coccidiosis costs the poultry industry an estimated 3 billion US dollars per year due to high mortality, poor growth and high medical costs^1–3^. Coccidiosis in chickens (and other animals) is caused by protozoa from the *Eimeria* genus (from the subclass Coccidia). Due to low efficiency and the disadvantages of current anti-coccidial drugs and vaccines^4–6^, edible plants and/or natural products are being considered as possible viable alternative substituents. However, despite considerable progress over recent years, safety, efficacy, and the mechanisms of the modes of action of edible plants and their compounds still require further study if they are to be considered a viable alternative to current anti-coccidial approaches^7^.

It has been reported that over 1200 plants have anti-protozoal activity^8,9^. So far, only about 20 herbal plants have been studied for anti-coccidial activities^4,10–18^. Among these, members of the *B*. *pilosa* (Asteraceae family) are used as foods and medicines worldwide^19^. The Food and Agriculture Organization of the United Nations and the Taiwan government list *B*. *pilosa* as a food staple^20^. We previously reported that *B*. *pilosa* manifests high anti-coccidial activity and low induction of drug resistance in *Eimeria* parasites^18,21^. However, the anti-coccidial mechanism underlying *B*. *pilosa* is not clear. Further, despite the discovery of over 200 compounds in *B*. *pilosa*^19^, the identities of its anti-coccidial compounds are unknown, which currently limits the commercial use of *B*. *pilosa* in the poultry industry.

In this study, we first tested the efficacy of *B*. *pilosa* against coccidiosis in chickens. Next, using a bioactivity-directed fractionation and isolation procedure, we identified the anti-coccidial compounds from this plant. In addition, we explored the mode of action of *B*. *pilosa* and its bioactive compounds using *in vitro* co-incubation with *E*. *tenella* oocysts and sporozoites. Finally, we also confirmed the anti-coccidal action of its bioactive compounds in chickens.

## Results

### Prophylactic efficacy of *B*. *pilosa* in chicken coccidiosis

**Figure 1:**
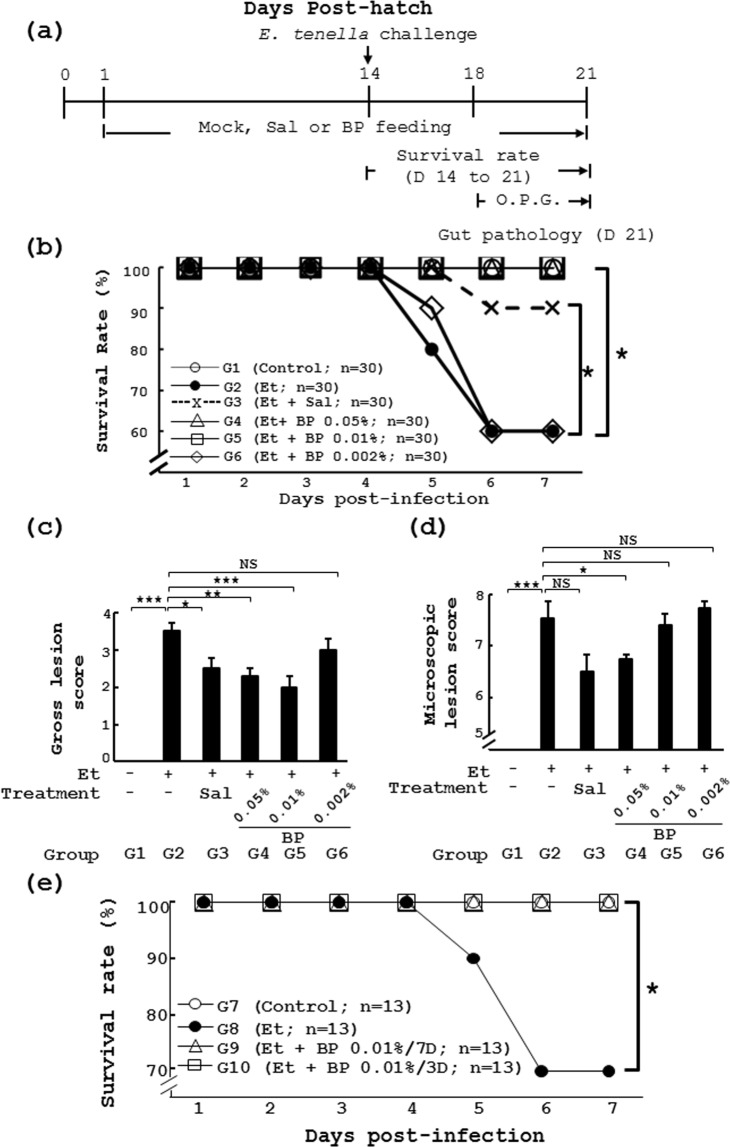
Preventive effect and minimum prophylactic duration of *B. pilosa* on coccidiosis in chickens. (**a**) The experimental protocol of the study. (**b**–**d**) Effect of *B*. *pilosa* on survival rate of chickens given *E*. *tenella* challenge. In Experiment 1, 6 groups of chicks had daily access to a diet containing vehicle, salinomycin (Sal) or different doses of *B*. *pilosa* (BP 0.05%, BP 0.01% and BP 0.002%). On day 14, chickens were administered with PBS or *E*. *tenella* sporulated oocysts (Et) by gavage. Survival rate was measured daily from day 1 to 7 post infection (**b**). Gross lesion score (**c**) and microscopic lesion (**d**) score were obtained from the grading of the cecal lesions of the same chicks as in Figure 1b. (**e**) In Experiment 2, 4 groups of chickens were used for the study. The chickens in Group 7 were fed with the standard diet from days 1 to 21 with *E*. *tenella* infection. Chicks were pre-administered the diet (Et, Group 8), from days 1 to 21, and the diet containing *B*. *pilosa* powder (0.01%), from days 11 to 14, for 3 days (Et + BP/3D, Group 9), and, from days 11 to 18, for 7 days (Et + BP/7D, Group 10), respectively. On day 14, the birds were orally infected with PBS or sporulated oocysts of *E*. *tenella*. The survival of the chicks was monitored from days 14 to 21. The number (*n*) of chicks in each group is indicated. *P* < 0.05 (*) was considered to be statistically significant.

Our previous publication showed that *B*. *pilosa* could protect chickens from *Eimeria tenella* infection^18^. With an eye to development of *B*. *pilosa* as a feed additive to prevent coccidiosis in chickens, here we explored its *in vivo* efficacy as measured by the minimum effective dose and minimum prophylactic duration. First, chickens were fed daily standard chicken feed from day 1 to day 21. The feed contains the commercial anti-coccidial chemical salinomycin or 0.05%, 0.01% and 0.002% *B*. *pilosa* powder as described in Fig. 1a. After challenging with *E*. *tenella*, chickens with standard feed had lower survival rate (60% in Group 2 (Et)) compared to the control group (100% in Group 1 (CTR)) (Fig. 1b). The challenged chickens with feed containing salinomycin had 90% survival rate in Group 3 (Et + Sal, Fig. 1b) as we expected. In contrast, the survival rates were 100%, 100%, and 60% for infected chickens with the feed containing *B*. *pilosa* at the doses of 0.05%, 0.01% and 0.002% (Groups **4** (Et + BP 0.05%), **5** (Et + BP 0.01%) and **6** (Et + BP 0.002%), Fig. 1b), respectively. Consistently, *B*. *pilosa* improved the body weight loss in chickens challenged with *E*. *tenella* (Table 1).The data suggest that the minimum effective dose of *B*. *pilosa* is 0.01% (100 ppm).

**Table 1:** Body weight gain (BWG) of chickens given standard diet with or without salinomycin and different doses of *B*. *pilosa* from days 1 to 21.

Group cage no. (chickens)	BWG(g)	*P*-value^a^	*P*-value^b^	BWG(g)	*P*-value^a^	*P*-value^b^
	Day 14-1	Day 14-1	Day 14-1	Day 21-1	Day 21-1	Day 21-1
G1 (n = 10) 3 (3, 3, 4)	126.73 ± 3.82			234.72 ± 3.4		
G2 (n = 10) 3 (3, 3, 4)	127.14 ± 3.21	>0.05		169.6 ± 8.81	<0.05	
G3 (n = 10) 3 (3, 3, 4)	126.35 ± 3.57	>0.05	>0.05	200.01 ± 5.91	<0.05	<0.05
G4 (n = 10) 3 (3, 3, 4)	120.66 ± 2.14	>0.05	>0.05	217.78 ± 5.37	<0.05	<0.05
G5 (n = 10) 3 (3, 3, 4)	125.51 ± 5.44	>0.05	>0.05	215.77 ± 6.88	<0.05	<0.05
G6 (n = 10) 3 (3, 3, 4)	130.93 ± 2.02	>0.05	>0.05	161.35 ± 4.53	<0.05	>0.05

The chickens in Experiment 1 were divided into Groups **1** to **6**. Group **1** (uninfected unmedicated control, CTR) and Group **2** (infected unmedicated control, Et) were given daily standard chicken diet from day 1 to day 21. Group **3** (Et + Sal) had daily access to a diet with salinomycin (Sal, 100 mg/kg diet). Group **4** (Et + BP 0.05%), Group **5** (Et + BP 0.01%), and Group **6** (Et + BP 0.002%) were fed daily with the diet containing *B*. *pilosa* powder at the indicated doses. The number (n) of chickens and cage number in each group and number of chickens in each cage are shown. Body weight gain (BWG): body weight on day T (14 or 21) – body weight on day 1. ^a^Nested ANOVA was used to determine the difference in chicken body weight gain (g) between infected groups (Groups 2–6) and uninfected unmedicated group (Group 1) and the data are presented by P value. ^b^Nested ANOVA was used to determine the difference in chicken body weight gain (g) between infected medicated groups (Groups 3–6) and infected unmedicated group (Group 2) and the data are presented by P value.

Consistently, the oocyst excretion from the chickens, expressed as oocysts per gram of feces (OPG), an indicator of *Eimeria* multiplication, was also evaluated. There were no oocysts in the feces of the unchallenged controls without medication (Group 1, Table 2). After *E*. *tenella* infection, the fecal oocyst excretion from days 4 to 7 was measured in all infected groups. The OPG in the infected unmedicated birds was between 4.18 × 10^4^ and 8.28 × 10^4^ (days 4 to 7 post-infection) (Group **2**, Table 2). As expected, the salinomycin-fed chickens with infection in Group **3** (Table 2) had significantly lower OPG than those in Group **2**. Similarly, the *B*. *pilosa*-fed chickens with infection in Group **4** (Et + 0.05% BP, Table 2) and Group **5** (Et + 0.01% BP, Table 2) had significantly fewer OPG than those in Group 2 as shown in Table 2. However, the chickens in Group **6** (Et + 0.002% BP, Table 2) had similar OPG to those in Group **2** (Table 2).

**Table 2:** Fecal oocyst excretion of chickens given standard diet with or without salinomycin and different doses of *B*. *pilosa* 4 to 7 days after challenge with *E*. *tenella*.

Group		Days post-infection			
		4	5	6	7
		Ln (OPG + 1)	Ln (OPG + 11)	Ln (OPG + 11)	Ln (OPG + 11)
CTR	G1 (n = 9)	0	0	0	0
Et	G2 (n = 9)	0	10.64 ± 7.65^a^	11.32 ± 9.16^a^	10.98 ± 10.07^a^
Et + Sal	G3 (n = 9)	0	8.98 ± 6.26^a,b^	9.58 ± 7.99^a,b^	9.23 ± 7.87^a,b^
Et + BP 0.05%	G4 (n = 9)	0	6.40 ± 4.76^a,b^	10.74 ± 8.06^a,b^	10.13 ± 8.40^a,b^
Et + BP 0.01%	G5 (n = 9)	0	6.15 ± 4.90^a,b^	10.85 ± 9.03^a,b^	10.36 ± 8.58^a,b^
Et + BP 0.002%	G6 (n = 9)	0	10.57 ± 7.99^a^	11.24 ± 9.69^a^	10.84 ± 9.63^a^

After challenge with *E*. *tenella* from day 3 to day 7, the oocysts per gram feces (OPG) of the same chickens from Table 1 in Experiment 1 were measured. The values (×10^4^) of chicken OPG in all the groups were transformed into Ln(OPG + 1) and the data was evaluated by ANOVA using the GLM procedure of the SAS system under a normal distribution. The number (n) of chickens in all the groups is shown.

^a^The P value (<0.05) is statistically significant in the chicken OPG between the infected groups (G2–6) and uninfected unmedicated group (G1) on the presented days.

^b^The P value (<0.05) is statistically significant in the chicken OPG between the infected medicated groups (G3–6) and infected unmedicated group (G2) on the presented days.

In parallel, the gross cecal lesion in the chickens with different diets was examined at post-infection day 7. Gross cecal lesion score is shown in Fig. 1c. The uninfected control chickens without medication (Group **1**, Fig. 1c) had no lesions in the ceca (score = 0). In contrast, the chickens without medication had more gross cecal lesions in gut 7 days after infection, as evidenced by a lesion score close to 4 (Group 2, Fig. 1c). Like salinomycin (Group **3**, Fig. 1c), *B*. *pilosa* at doses of 0.05% and 0.01%, but not 0.002%, significantly reduced cecal damage in challenged chickens (Groups **4** to **6**, Fig. 1c) as shown by the gross lesion scores of 2.0 to 3.0 and microscopic lesion score of 6.8 to 7.7 (Groups **4** to **6**, Fig. 1d).

Further, we tried out the minimum prophylactic duration of *B*. *pilosa* in chickens. We found that the preventive use of *B*. *pilosa* at the dose of 0.01%, once a day for 3 and 7 days, could fully protect chickens from coccidiosis as evidenced by survival rate of chickens (Groups **7** to **10**, Fig. 1e). These data suggest that the minimum prophylactic duration of *B*. *pilosa* is as short as 3 days.

Overall, *B*. *pilosa* showed a high level of anti-coccidial efficacy, superior to that of the commercial anti-coccidial chemical, salinomycin.

### *B*. *pilosa* suppresses sporulation and invasion of *E*. *tenella*

**Figure 2:**
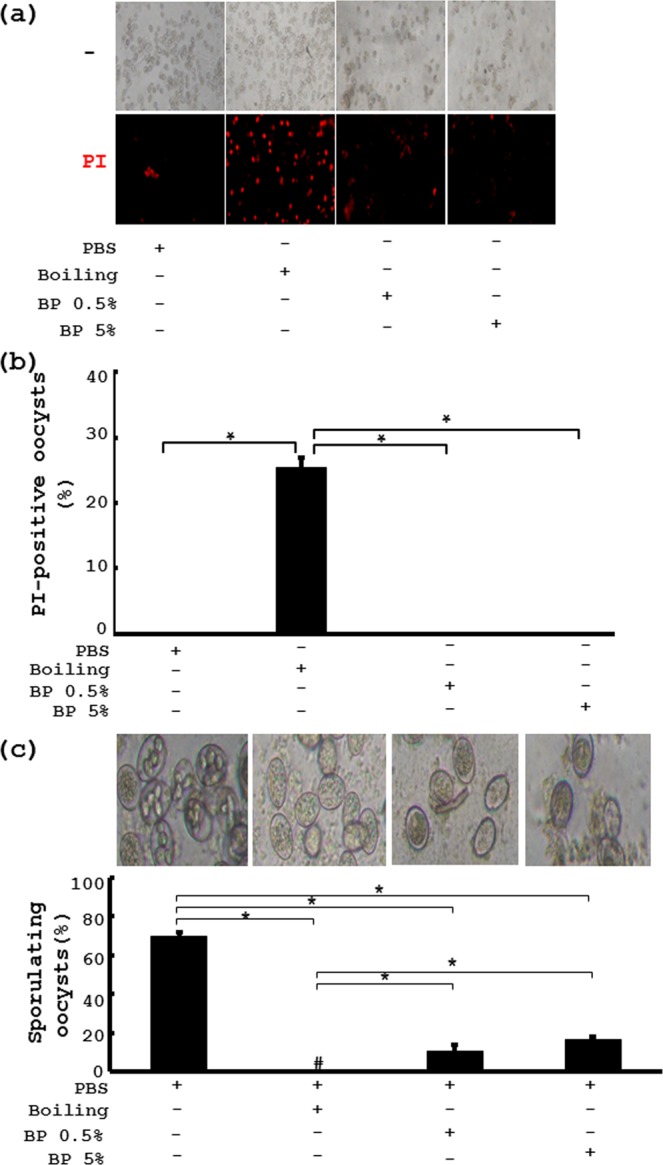
*In vitro* effect of *B*. *pilosa* on *E*. *tenella* oocyst viability and sporulation. (**a**) The oocysts were pre-treated with PBS, boiling and *B*. *pilosa* at 5% and 0.5% for 48 h. After PI staining, the oocyst viability was examined using a microscope. (**b**) Percentage of PI-positive oocysts, presented as mean  ±  SE, was plotted into bar graphs. (**c**) The oocysts were induced to sporulate by potassium dichromate for 2 days. The percentage of sporulating oocysts was counted using microscopy (top panel) and plotted into bar graphs (bottom panel).

**Figure 3:**
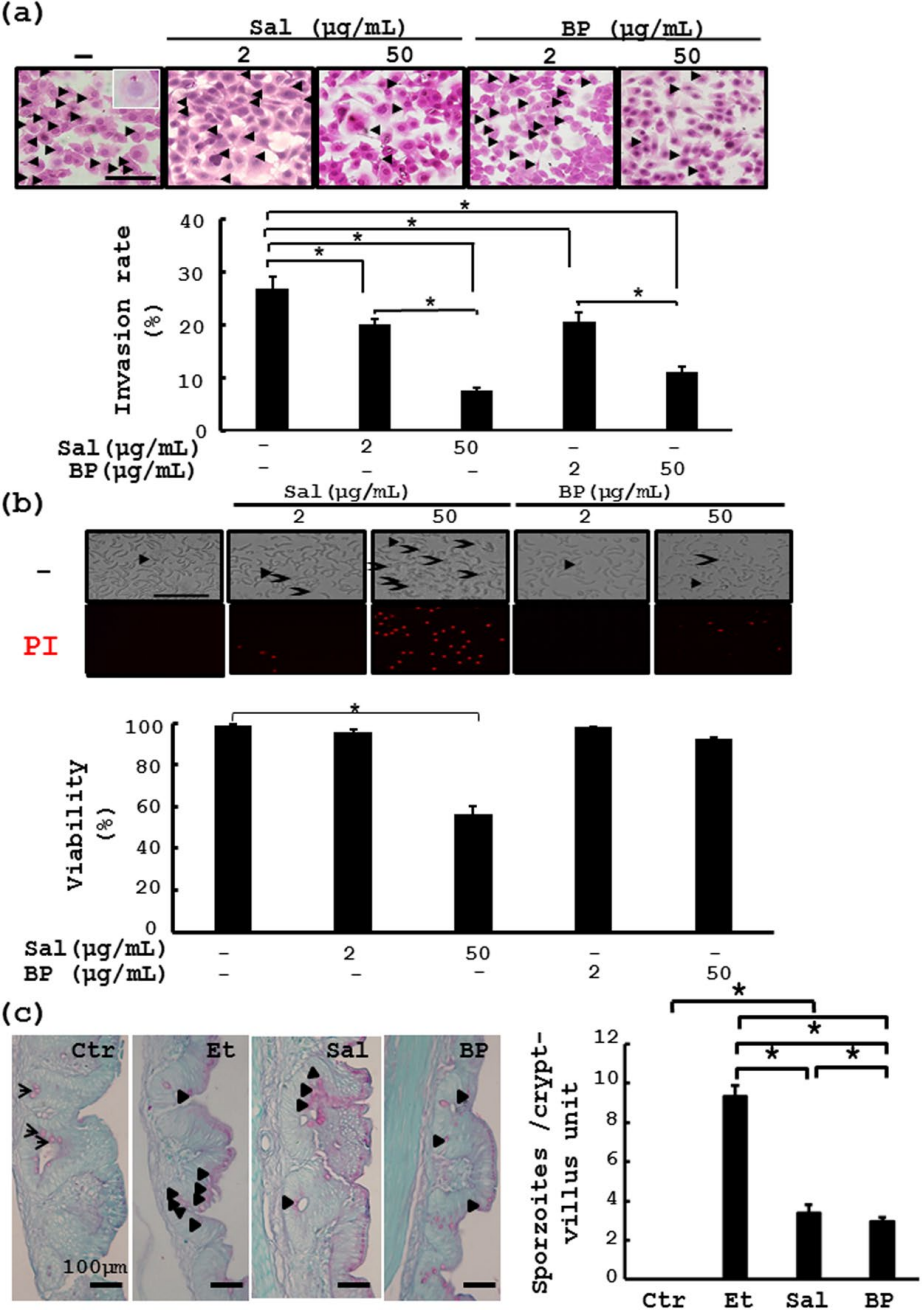
*In vitro* and *in vivo* effect of *B*. *pilosa* on *E*. *tenella* sporozoite invasion and viability. (**a**) MDBK cells were incubated with PBS vehicle, salinomycin (Sal) and *B*. *pilosa* powder (BP) at the indicated doses for 0.5 h. The sporozoites were added to the cells for an additional 4 h. After extensive washing, the cells were stained with hematoxylin and eosin and counted (top panel). The invasion percentage (%) was plotted into bar graphs (bottom panel). (**b**) The sporozoites were incubated with PBS, salinomycin (Sal) and *B*. *pilosa* powder (BP) at the indicated doses for 4.5 h. Following propidium iodide (PI) staining, the cells were photographed (top panel) and the viability (%) of the sporozoites was determined and plotted into bar graphs (bottom panel). (**c**) The *in vivo* entry of *E*. *tenella* sporozoites into chicken ceca in the chickens of Group **17** (CTR), Group **18** (Et), Group **19** (Et + Sal), Group **20** (Et + 0.01% BP) in Experiment 4 were analyzed. The number of the sporozoites per crypt-villus unit in chicken ceca was counted. Goblet cells (arrows) and sporozoites (arrow heads). *P* < 0.05 (*) was considered to be statistically significant.

To tease out the mode of action of *B*. *pilosa* on coccidiosis, we first examined the direct killing activity of *B*. *pilosa* in *E*. *tenella* oocysts. As expected, boiling treatment, as a positive control, could effectively kill the oocysts as demonstrated by PI staining (Fig. 2). However, *B*. *pilosa* at high doses (5% and 0.5%) failed to kill the oocysts (Fig. 2). Next, we tested the effect of *B*. *pilosa* on the sporulation of *E*. *tenella* oocysts. Seventy percent of the oocysts were able to sporulate in the *in vitro* culture (PBS, Fig. 2c). However, boiling treatment completely stopped this sporulation (Boiling, Fig. 2c). In sharp contrast, in the presence of *B*. *pilosa* at 0.5% to 5%, less than 20% of the oocysts sporulated (BP, Fig. 2c). Finally, we checked the effect of *B*. *pilosa* on the entry of *E*. *tenella* sporozoites into MDBK cells. As reported in a previous publication^22^, the sporozoites could invade into 27% of the cells (Fig. 3a). Salinomycin at the doses of 2 and 50 μg/ml, decreased this invasion to 20% and 8%, respectively. In contrast, *B*. *pilosa*, at the doses of 2 and 50 μg/ml, also reduced the invasion to 21% and 11%, respectively (Fig. 3a). In contrast, viability assay showed that salinomycin induced dose-dependent death of the sporozoites (Fig. 3b). However, *B*. *pilosa*, failed to induce death of the sporozoites or MDBK cells at the indicated dosages (Figs 3b and S1). These data demonstrate that, unlike salinomycin, *B*. *pilosa* inhibited oocyst sporulation and sporozoite invasion but did not directly kill oocysts and sporozoites. Moreover, the histochemical data on the ceca of chickens infected with *E*. *tenella* sporozoites which were pre-treated with salinomycin and *B*. *pilosa* showed that like *in vitro* invasion assay, *B*. *pilosa* inhibited the *in vivo* entry of the sporozoites into gut cells in chickens (Fig. 3c). Consistently, we also found that *B*. *pilosa*, reduced the percentage and size of the second-generation schizonts (Fig. S2a–c) and the number of fecal oocysts (Fig. S2d). Collectively, these data clearly demonstrate that *B*. *pilosa* interfered with the life cycle of *E*. *tenella* at oocyst sporulation, sporozoite invasion and schizont maturation.

### Cytopiloyne, the most active compound present in *B*. *pilosa*, suppresses sporozoite invasion and coccidiosis in chickens

To better understand the anti-coccidial mechanism of *B*. *pilosa*, we next turned our attention to identifying the anti-coccidial compounds present in *B*. *pilosa*, and their anti-coccidial action. First, we combined invasion assays and phytochemistry to identify active phytochemicals from *B*. *pilosa* based on a bioactivity-guided fractionation and isolation strategy (Fig. S3). Three polynes, 2-β-_D_-glucopyranosyloxy-1-hydroxytrideca-5,7,9,11-tetrayne (Compound **1**, also named cytopiloyne, 0.021%), 2-β-_D_-glucopyranosyloxy-1-hydroxy-5(E)-tridecene-7,9,11-triyne (Compound **2**, 0.018%), and 3-β-_D_-glucopyranosyloxy-1-hydroxy-6(E)-tetradecene-8,10,12-triyne (Compound **3**, 0.013%) were identified from this plant. Their structures were elucidated and confirmed using a UV spectrophotometer (Fig. S4), mass spectroscope (Fig. S5) and nuclear magnetic resonance instrument (data not shown).

**Figure 4:**
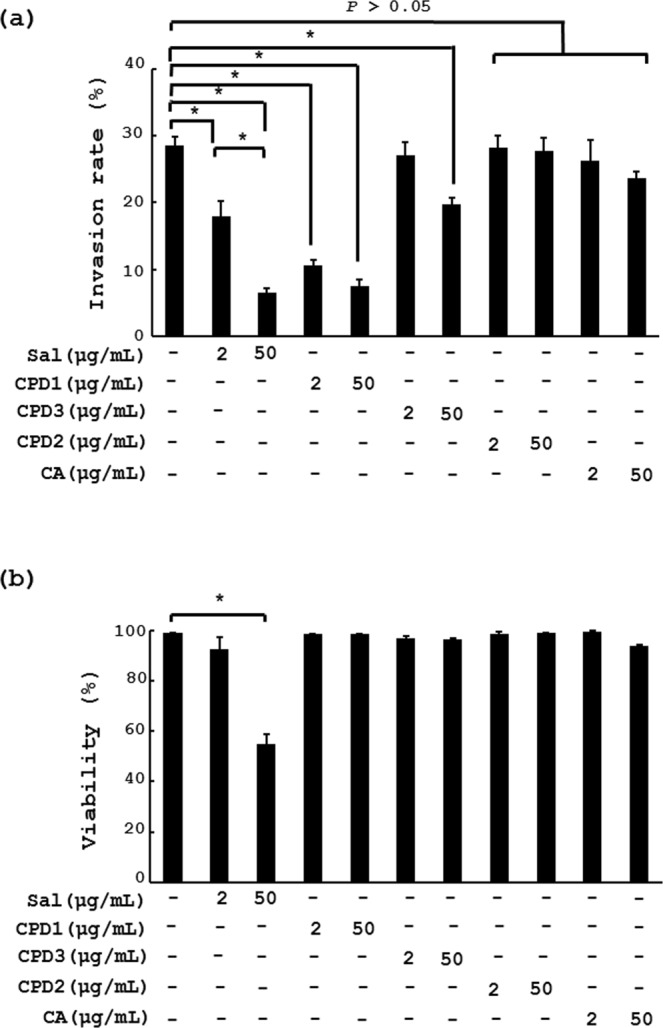
*In vitro* effect of phytochemicals extracted from *B*. *pilosa* on *E*. *tenella* sporozoite invasion and viability. (**a**) MDBK cells were pre-incubated with PBS vehicle, salinomycin (Sal), 3 polyynes (CPD1, CPD2 and CPD3) and chlorogenic acid (CA) at the indicated doses for 0.5 h, followed by additional incubation with sporozoites using the same procedure as in Fig. 3. The invasion percentage (%) is presented on a bar graph. (**b**) The same sporozoites as (**a**) were examined for viability. Their viability (%) was determined and plotted into a bar graph (**b**). *P* < 0.05 (*) was considered to be statistically significant.

In parallel, invasion assays were conducted to evaluate the anti-coccdial activity of the 3 polyynes. As expected, salonomycin, used as a positive control, dose-dependently inhibited the invasion of *E*. *tenella* spopozoites into MDBK cells (Sal, Fig. 4a). A phenolic compound, chlorogenic acid (CA, Fig. 4a), used as a negative control, did not affect this invasion. In contrast, cytopiloyne (CPD **1**, Fig. 4a) exhibited the most potent inhibition of the entry of sporozoites into MDBK cells in comparison with the other two polyynes (CPD **2** and **3**, Fig. 4a) and an inactive phenolic, chlorogenic acid (CA, Fig. 4a). This inhibition was not due to the cytotoxicity of cytopiloyne since cytopiloyne failed to kill sporozoites in a direct way (Fig. 4b). Similar to the anti-coccidial mechanism of *B*. *pilosa*, the action of cytopiloyne against *E*. *tenella* could be attributed to the sporozoite invasion into gut cells, but not direct killing of sporozoites (Fig. 4b) nor suppression of oocyst sporulation (data not shown). All these polyynes and cholorogenic acid did not affect MDBK cell viability (Fig. S1).

**Figure 5:**
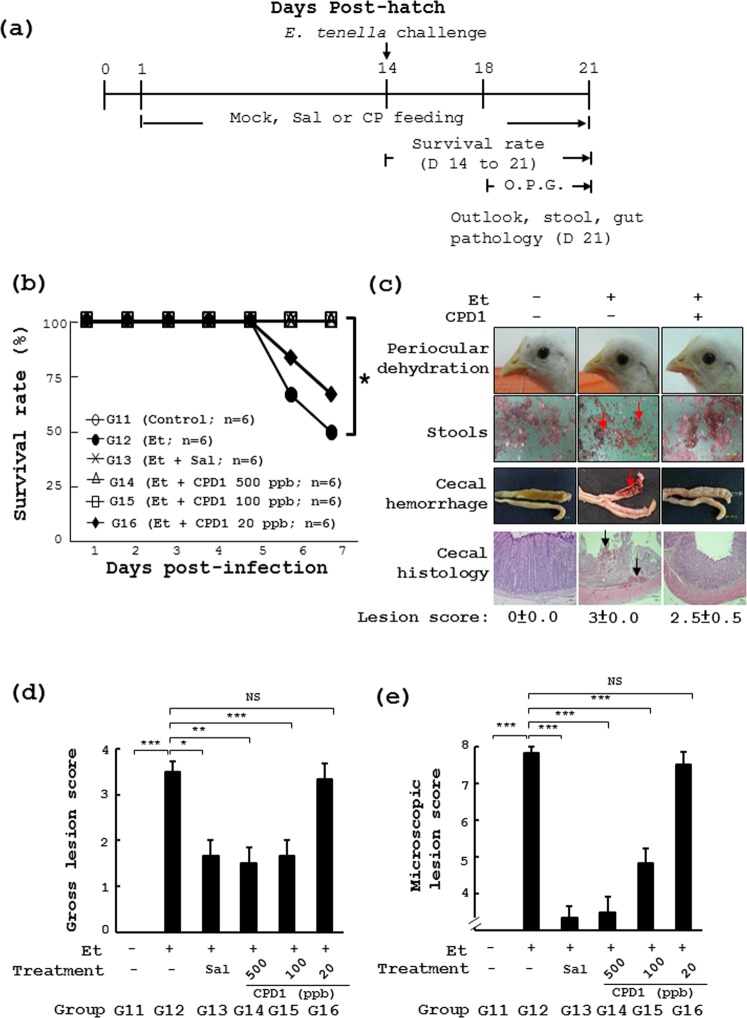
Preventive effect of cytopiloyne on coccidiosis *in vivo*. (**a**,**b**) The experimental protocol of the study (**a**). The same procedure as Fig. 1 except that cytopiloyne (CPD1) was used in the study (**b**). In Experiment 3, 6 groups of chicks had daily access to the standard diet (CTR) or a diet containing cytopiloyne (Et + CPD1) at the indicated dose. On day 14, chickens were infected with PBS or sporulated *E*. *tenella* oocysts (Et) by gavage. Survival rate was measured daily from day 1 to 7 post infection. (**c**) Periocular dehydration, stools and gut pathology were measured. The number (*n*) of chicks in each group is indicated. (**d**,**e**) Gross lesion score and microscopic lesion score were obtained from the grading of the cecal lesions of the same chicks as in (**b**). The number (*n*) of chicks in each group is indicated. *P* < 0.05 (*) was considered to be statistically significant.

We also checked the anti-coccidial effect of the most active polyyne, cytopiloyne, in chickens. Chickens received daily standard chicken feed (day 1 to day 21) containing salinomycin (Sal, Fig. 5a,b) or cytopilloyne at 500 ppb, 100 ppb and 20 ppb (CPD1, Fig. 5a,b). After *E*. *tenella* challenge, the survival rate dropped from 100% (Group 11) to ~50% (Group 12) in the chickens with standard feed (Fig. 5a,b). However, the survival rate of infected chickens with feed containing cytopiloyne at 500 ppb, 100 ppb and 20 ppb was 100%, 100% and 67%, respectively (Groups **14** to **16**, Fig. 5b). In addition, chickens infected with *E*. *tenella* also showed periocular dehydration, bloody stools, and cecal bleeding/damage (Group **12**, Fig. 5c). In sharp contrast, similar to the uninfected controls (Group **11**, Fig. 5c), birds fed cytopiloyne at 500 ppb showed no sick bird signs (periocular dehydration, Fig. 5c) or bloody stools (Fig. 5c). Accordingly, cytopiloyne dose-dependently reduced cecal bleeding (Fig. 5c) and damage (Fig. 5c–e) and fecal oocyst counts (OPG, Table 3). Taking these results together, we conclude that cytopiloyne exerted great anti-coccidial activity in chickens via regulation of sporozoite sporulation and invasion.

**Table 3:** Fecal oocyst excretion of chickens given standard diet with or without salinomycin and different doses of cytopiloyne (CPD1) 4 to7 days after challenge with *E*. *tenella*.

Group		Day post-infection			
		4	5	6	7
		Ln (OPG+1)	Ln (OPG+1)	Ln (OPG+1)	Ln (OPG+1)
CTR	G11 (n = 3)	0	0	0	0
Et	G12 (n = 3)	0	8.03 ± 4.21^a^	11.45 ± 9.37^a^	11.38±9.56^a^
Et+Sal	G13 (n = 3)	0	6.91 ± 5.30^a,b^	9.69 ± 9.55^a,b^	9.32 ± 9.19^a,b^
Et+CPD1 500 ppb	G14 (n = 3)	0	6.40 ± 4.76^a,b^	9.69 ± 9.55^a,b^	9.33 ± 9.19^a,b^
Et+CPD1 100 ppb	G15 (n = 3)	0	6.15 ± 4.90^a,b^	10.60 ± 7.48^a,b^	10.53 ± 7.35^a,b^
Et+CPD1 20 ppb	G16 (n = 3)	0	8.01 ± 4.76^a^	10.48 ± 10.34^a^	10.30 ± 10.19^a^

After challenge with *E*. *tenella* from day 3 to day 7, the oocysts per gram feces (OPG) of the same chickens from Fig. 3c in Experiment 3 were measured. The values (×10^4^) of chicken OPG in all the groups were transformed into Ln(OPG + 1) and the data was evaluated by ANOVA using the GLM procedure of the SAS system under a normal distribution. The number (n) of chickens in all the groups is shown.

^a^The P value (<0.05) is statistically significant in the chicken OPG between the infected groups (G12–16) and uninfected unmedicated group (G11) on the presented days.

^b^The P value (<0.05) is statistically significant in the chicken OPG between the infected medicated groups (G13–16) and infected unmedicated group (G12) on the presented days.

## Discussion

Coccidiosis is a bane to the poultry industry causing considerable economic loss. Misuse and abuse of current anti-coccidial drugs in poultry farming has raised public concerns about food safety. Edible herbs are emerging as an alternative approach to treat coccidiosis in chickens^4,8,17^. However, the use of medicinal herbs in coccidiosis is limited by the complexity of constituent phytochemicals and unknown mechanisms. Our previous publication demonstrated that *B*. *pilosa* has promising efficacy and safety^18,23^. Here, we extended our study to explore the minimum effective dose and prophylactic duration, identify the active compound(s) and elucidate the mechanism of *B*. *pilosa* and its active compounds. The results of this study will aid the development *B*. *pilosa* as an anti-coccidial phytogenic and medicine prior to commercial use in chickens.

In terms of anti-coccidial efficacy, we proved that the effective dose of *B*. *pilosa* could be as low as 0.01% under our experimental conditions (Fig. 1). In addition, 3-day administration of 0.01% *B*. *pilosa* was good enough to achieve anti-coccidial prevention (Fig. 1e). Of note, some parameters may affect the efficacy of *B*. *pilosa*; combinatorial infection with different *Eimeria* species, titer and virulence of *Eimeria* species, and chicken genetics^24,25^.

*Eimeria* species have a complex life cycle that starts when the sporulated oocysts are swallowed by chickens. The grinding action of the gizzard coupled to the enzymatic action in the gut lead to sporozoite release. The sporozoites develop into merizoites, followed by gametocytes, zygocytes and oocytes^26^. In this work, we illustrated that *B*. *pilosa* interfered with oocyst sporulation (Fig. 2c) and sporozoite invasion into cells (Fig. 3a) but not the viability of oocysts (Fig. 2a,b) and sporozoites (Fig. 3b). The histochemical staining of chicken ceca also showed that *B*. *pilosa* decreased the percentage of schizonts and their size (Fig. S2a–c) and the number of fecal oocysts (Fig. S2d), leading to production of precocious oocysts. All these data support the notion that *B*. *pilosa* interfered with the life cycle of *E*. *tenella* at the stages of sporogony, merogony, and, probably, gametogony. This anti-coccidial mode of action has an advantage over chemical anti-coccidials. Namely that *B*. *pilosa* may impair but not completely kill *Eimeria* progeny which, may in turn serve as a vaccine to boost host immunity to coccidiosis. Besides, the data on the intervention of sporozoite invasion by *B*. *pilosa* are consistent with a decrease in the shedding of fecal oocysts and survival rate in experimental chickens (Table 2). This work also demonstrates the feasibility of *B*. *pilosa* as a veterinary medicine for controlling coccidiosis in chickens.

Identification of active compound(s) from plants is a key challenge to developing herbal applications for medical purposes. Using a bioactivity-directed strategy, here we found that cytopiloyne inhibited the entry of sporozoites into cells more effectively than salinomycin, Compound **3** and Compound **2** (Fig. 4a). This result confirmed that cytopiloyne is the most active polyyne against coccidiosis. Of note, *B*. *pilosa* at 100 ppm and cytopilyne at 100 ppb fully protected against coccidiosis in chickens (Figs 1 and 5), suggesting that cytopiloyne was 1000 times more effective against coccidiosis than *B*. *pilosa*. Coincidently, the percentage of three polyynes in *B*. *pilosa* was 0.52‰, implying that polyynes are the major active phytochemicals of *B*. *pilosa*, although we cannot rule out the existence of other active compound(s) (Figs 4, 5 and S3). In a similar manner to *B*. *pilosa* extract (Figs 1–3), cytopiloyne exerted its anti-coccidial activities via suppression of sporozoite sporulation (data not shown) and invasion into cells (Figs 3c and 4a,b). Obviously, *B*. *pilosa* suppresses coccidiosis in chickens via interference with the life cycle of *Eimeria* (Figs 3, 4 and S2), but not *via* direct chemical destruction (Figs 3b and 4b). Therefore, this study provides the first evidence of the mechanism of *B*. *pilosa* in control of coccidiosis, a key step in research and development of in-feed additives and medicines against coccidiosis.

*B*. *pilosa* has been reported to modulate immune responses in animals^27–33^. As far as chicken immunity to coccidiosis is concerned, intestinal T cells have been reported to play a major role in host protection against coccidiosis in chickens^34^. We examined the impact of *B*. *pilosa* on T cells using a chicken Affymetrix genechip. The genome-wide study found that *B*. *pilosa* influenced the expression of 540 genes with a more than 1.5-fold increase (176 genes) or 2-fold decrease (364 genes) in T cells (data not shown). Among 540 genes, 100 genes were functionally known and selected for heatmap analysis (Fig. S2a). IFNγ, an anti-coccidial immunomodulator, was up-regulated by *B*. *pilosa* during *E*. *tenella* infection (Fig. S6b). Under non-infection conditions, *B*. *pilosa* did not boost IFNγ production (Fig. S6b). The data are consistent with the literature stating that IFN-γ expression was significantly increased in cecal tonsils which are an important component of the host immunity against coccidiosis^35,36^. However, whether the polyynes can increase IFN-γ needs to be ascertained.

Here, we assessed the efficacy, minimum effective dose and minimum prophylactic duration of *B*. *pilosa* for treating coccidiosis in chickens as evidenced by reducing mortality, oocyst excretion, intestinal lesions and body weight gain. In parallel, we identified three polyynes as active compounds present in *B*. *pilosa* using a bioactivity-guided approach. Among the polyynes, cytopiloyne was the most active compound in *B*. *pilosa*. Furthermore, we demonstrated that *B*. *pilosa* and cytopiloyne exert their anti-coccidial action via intervention with the protozoan life cycle and augmenting chicken immunity. In conclusion, this study demonstrates the anti-coccidial effects and mechanism of *B*. *pilosa* and its active compounds in chickens.

## Methods

### Preparation and analysis of *B*. *pilosa* and polyynes

The processing and analysis of *B*. *pilosa* were performed as previously published^18^. Briefly, the whole plant was authenticated by Dr. Kuo-Fang Chung (Academia Sinica Herbarium), collected and pulverized. For compound isolation and identification, *B*. *pilosa* was extracted with methanol and partitioned into different fractions, followed by compound isolation and identification using high pressure liquid chromatography^37^ unless indicated otherwise. Using an invasion assay-guided fractionation and isolation strategy, active polyynes were isolated and identified by spectroscopic methods as described elsewhere.

### Preparation and sporulation of *E*. *tenella* oocysts

As previously described^18^, the *E*. *tenella* strain Et C1 was amplified and used throughout the study. The oocysts were collected from fresh feces of chickens, followed by sporulation with potassium dichromate.

### Poultry husbandry, feed formula and oral infection of *E*. *tenella*

One-day-old uninfected Lohmann female chicks were obtained from a local hatchery. For efficacy study of *B*. *pilosa*, the chickens were randomly divided into 6 groups. They had *ad libitum* access to diets and water in the experiments. In Experiment 1, Group **1** (uninfected unmedicated control, CTR) and Group **2** (infected unmedicated control, Et) received daily standard chicken diet from day 1 to day 21. Group **3** (Et + Sal) were given a daily diet containing salinomycin (Sal, 60 mg/kg diet). Group **4** (Et + BP 0.05%), Group **5** (Et + BP 0.01%), and Group **6** (Et + BP 0.002%) were fed daily with a diet containing *B*. *pilosa* powder at the dose of 0.05% (0.5 g BP/kg diet), 0.01% (0.1 g BP/kg diet) or 0.002% (0.02 g BP/kg diet), respectively. In Experiment 2, to test the minimum prophylactic duration of *B*. *pilosa* powder, 4 groups of chickens (Groups **7** to **10**) were fed with a standard diet or a diet containing *B*. *pilosa* powder (0.01%) for the indicated time periods prior to *E*. *tenella* challenge, on day 14. In Experiment 3, which was an efficacy study of Compound **1** (cytopiloyne, CP), the chickens were randomly divided into 6 groups. The chickens in Group **11** (CTR), Group **12** (Et), Group **13** (Et + Sal), Group **14** (Et + 500 ppb CP), Group **15** (Et + 100 ppb CP) and Group **16** (Et + 20 ppb CP) were fed daily with a standard diet and a diet containing salinomycin (Sal, 60 mg/kg diet) and CP (500, 100 and 20 μg/kg diet).

Chickens were challenged with *E*. *tenella* on day 14. Control chickens in Groups **1**, **7** and **11** were given 2 ml of phosphate buffered saline (PBS) and those in Groups **2** to **6**, **8** to **10**, and **12** to **16** were challenged with *E*. *tenella* sporulated oocysts (1 × 10^4^) on day 14. Survival rate, gut pathology, stool, and/or sick bird appearance were observed daily unless indicated otherwise in each group. Based on the study by Daszak *et al*., initial invasion of the fold tip of cecum occurs at ~4 hr post infection, when sporozites of *E*. *tenella* invade enterocytes and migrate through the connective tissue into the crypt epithelium^38^. In Experiment 4, to test the entry of *E*. *tenella* sporozoites into chicken guts, the chickens were randomly divided into 4 groups. The chickens in Group **17** (CTR), Group **18** (Et), Group **19** (Et + Sal), Group **20** (Et + 0.01% BP) were fed daily with a standard diet. On day 14, the chickens in Group **17** (CTR) were given 2 ml PBS and those in Group **18**, **19** and **20** were challenged with a dose (1 × 10^4^) of *E*. *tenella* and *E*. *tenella-*treated with salinomycin and 0.01% *B*. *pilosa* powder (0.1 g BP/kg diet). The chickens were sacrificed 4 hr post infection. The chicken ceca were fixed with formaldehyde, microtomized and stained with a periodic acid-Schieff kit as published^39^. All chickens in the study were complied with according to the guidelines and were approved by Institutional Animal Care and Use Committee (IACUC) of the National Chung Hsing University (permit number: 100–60).

### Evaluation of survival rate, oocyst numbers, and gut lesions in animals

Survival rate and chicken appearance were observed daily as described previously^18,23^. The body weight of all the birds in the cages were measured on days 1, 7, 14 and 21 after hatching. Fecal samples were collected daily, from day 3 to 7 post infection, weighed and counted. Fecal oocyst number, expressed as oocysts per gram of feces (OPG), was obtained from the average of 3 counts of each sample. On day 14 post infection, each group of chickens was sacrificed and their ceca were collected. Macroscopic (gross) and microscopic lesion scores were calculated as described in our previous publication^18,23^.

### Invasion assay, viability test and propidium iodide (PI) staining of *E*. *tenella* sporozoites

Madin–Darby bovine kidney (MDBK, ATCC CCL-22) cells were grown in DMEM containing 10% fetal bovine serum and supplements. The cells were seeded at a density of 2 × 10^5^ cells/well onto glass cover slips in 24 wells. One day later, the cells were incubated with DMEM medium containing salinomycin (Fluka), plant extracts and phytochemicals at the indicated doses for 0.5 h. Fresh sporozoites (2 × 10^5^) were added to the cells for an additional 4 h. After extensive PBS washing, the cells were fixed and stained with hematoxylin and eosin (Sigma). Photographs were taken with a microscope. Invasion percentage (%) was obtained by the formula, 100% × (the number of cells invaded by sporozoites/total cell number)^22^. For the viability test, the *E*. *tenella* sporozoites were incubated with plant extract, phytochemicals and salinomycin for 4.5 h. Microscopy was used to distinguish life and death in sporozoites. Survival rate (%) was obtained by the normalization of the dead cell number by total cell number multiplied by 100%. For sporulation assay, the *E*. *tenella* oocysts were pre-treated with PBS, boiling (100 °C for 30 min) and plant extracts at the indicated doses for 48 h. The oocysts were incubated with 2% potassium dichromate for 2 days before sporulation. The percentage of sporulating oocysts (%) was counted. For PI staining, the *E*. *tenella* oocysts underwent PBS (1 h), boiling treatment (100 °C for 30 min) or incubation with *B*. *pilosa* extracts at the indicated doses for 1 h. The oocysts were stained with PI. After PBS washing, the oocysts were examined using a microscope^15^.

### Statistical analysis

Data from each group of chickens are presented as mean ± standard error (SE). The survival rate between treatment groups and control groups were analyzed using Pearson’s chi square test. The body weight gain of the factors group and cage group were analyzed by two way ANOVA using the GLM procedure of the SAS System. Data of the excreted oocyst was transformed into ln(x + 1) and subjected to ANOVA using the GLM procedure of the SAS System under a normal distribution. Chi-square test was used to value lesion scores after multinomial transformation. Actual P values of all experiments are presented.

## Supplementary information

Dataset 1
