# Supplementary material for: Anti-coccidial properties and mechanisms of an edible herb, Bidens pilosa, and its active compounds for coccidiosis
Source: Sci Rep. 2019 Feb 27;9:2896. doi: 10.1038/s41598-019-39194-2 (PMC6393484; doi:10.1038/s41598-019-39194-2)
Supplement: Supplementary file 1 — Dataset 1 [file 41598_2019_39194_MOESM1_ESM.pdf]

Anti-coccidial properties and mechanisms of an edible herb, *Bidens pilosa*, and its active compounds for coccidiosis

Wen-Chin Yang[1,2†], Cheng-Ying Yang[3†], Yu-Chuan Liang[1], Chu-Wen Yang[4], Wei-Qun Li[3], Chih-Yao Chung[3], Meng-Ting Yang[1,5,6], Tien-Fen Kuo[1], Chuen-Fu Lin[7], Chih-Lung Liang[8], Cicero Lee-Tian Chang[3*]

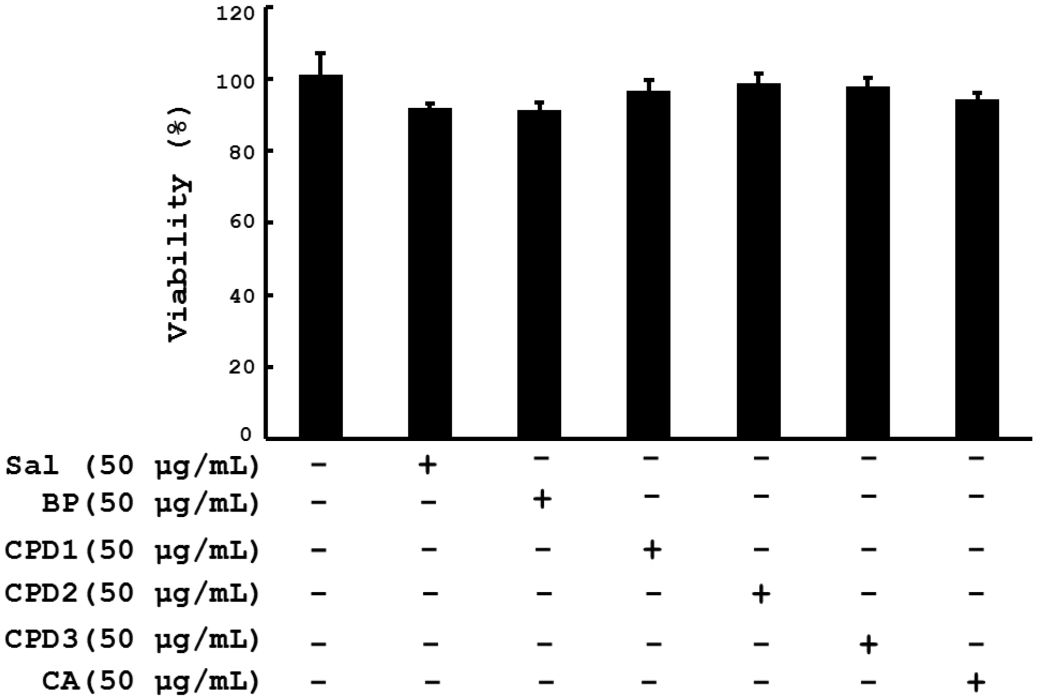

Fig. S1. Effect of *B. pilosa* and its compounds on cell culture. MDBK cells were incubated with salinomycin (Sal), *B. pilosa* (BP), CPD1, CPD2, CPD3 or chlorogenic acid (CA) at 50 µg/ml for 24 hr. Their viability was determined.

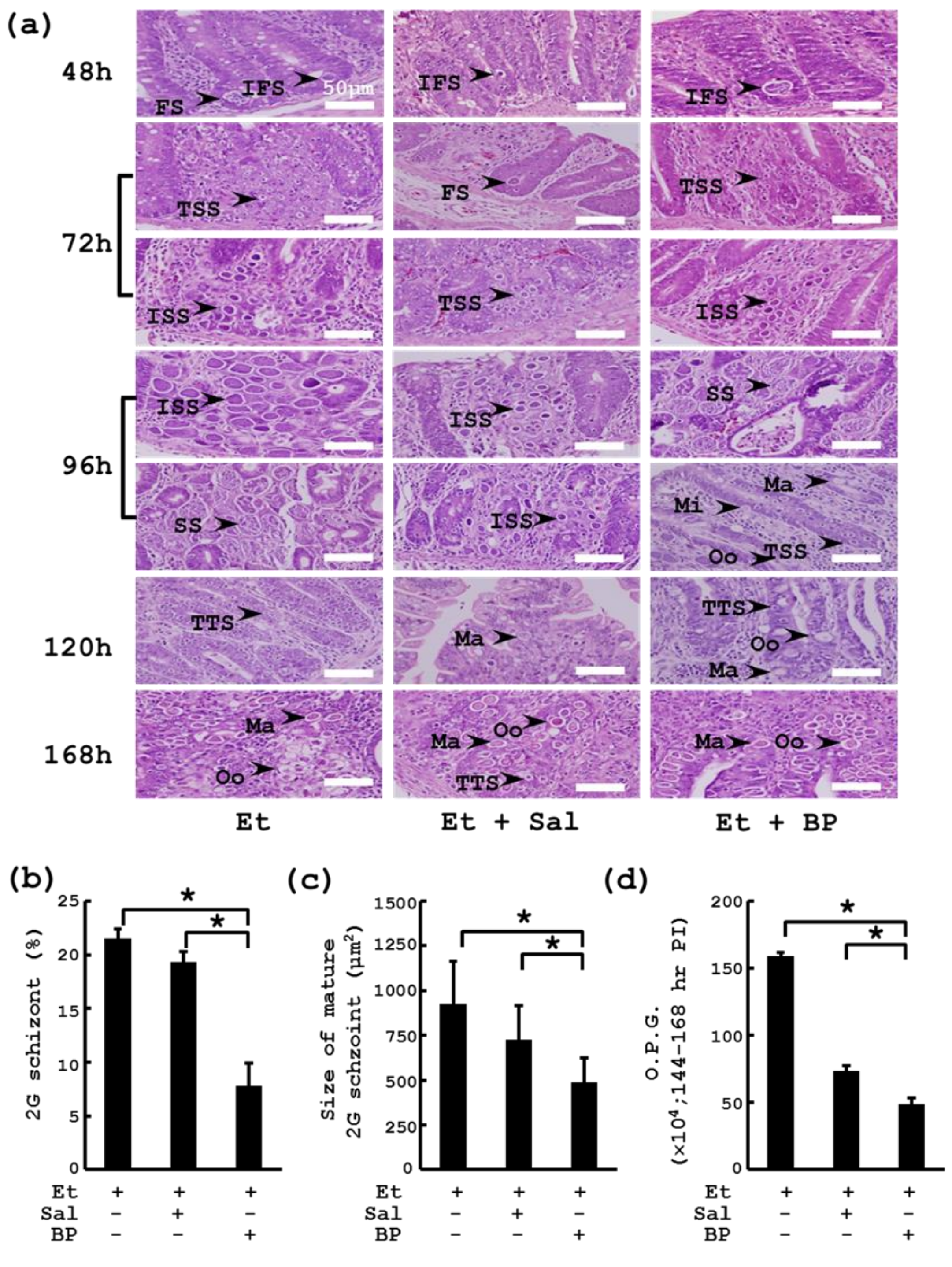

Fig. S2. Effect of *B. pilosa* on different stages of the life cycle of *E. tenella*. Three groups of chickens were treated according to the same protocol as those in Groups 2 (infected unmedicated control, Et), 3 (Et + Sal), and 5 (Et + BP 0.01%) in Experiment 1. The birds were sacrificed 48 to 168 hr post infection. The ceca underwent histochemical staining with hematoxylin and eosin. (a) Photographs of the ceca were taken under a microscope. Bar: 50 μm. Different stages of the life cycle of *E. tenella* are described below. IFS: immature first-generation schizont; FS: first-generation schizont; TSS: tropozoite of second-generation schizont; ISS: immature

second-generation schizont; SS: second-generation schizont; TTS: tropozoite of third-generation schizont or immature gametocyte; TS: third-generation schizont; Ma: macrogametocyte; Mi: microgametocyte; and Oo: oocyst. (b-c) Image J was used to analyze the percentage (b) and size (c) of second-generation schizonts in the ceca of chicken groups 120 hr after *E. tenella* infection. (d) Fecal oocyst per gram of feces (OPG) from chicken groups 144 to 168 hr after the *E. tenella* infection was counted.

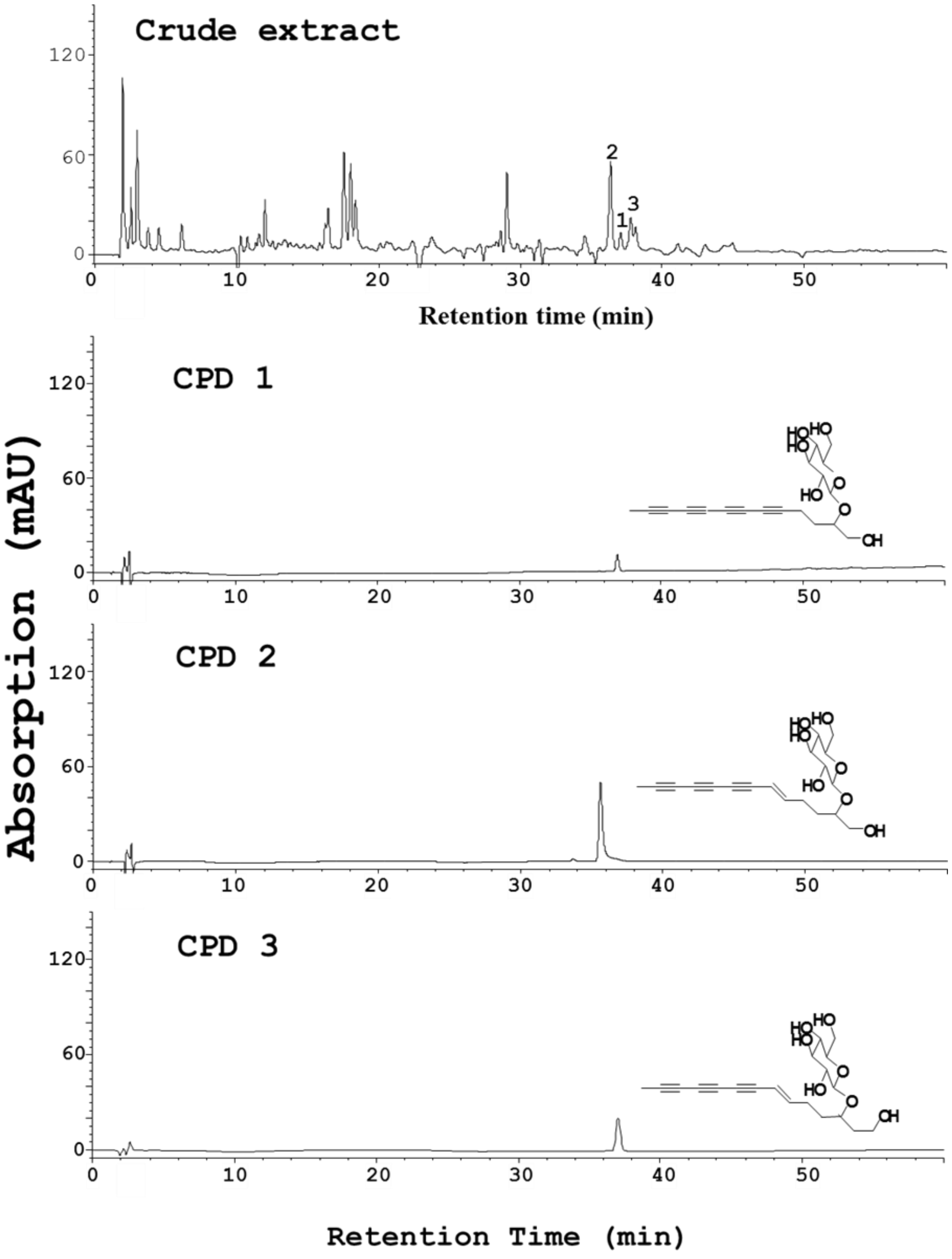

Fig. S3. High performance liquid chromatography profiles of *Bidens pilosa* extracts and three polyynes. The *B. pilosa* extracts and their three active polyynes underwent high performance liquid chromatography (HPLC) with photodiode detection at 254 nm. UV spectra of peaks 1 to 3 in the extract and compounds **1** to **3** of *B. pilosa* are indicated. Compound **1:** 2-β-D-glucopyranosyloxy-1-hydroxytrideca-5,7,9,11-tetrayne (cytopiloyne); Compound **2:** 2-β-D-glucopyranosyloxy-1-hydroxy-5(E)-tridecene-7,9,11-triyne; Compound **3:** 3-β-D-glucopyranosyloxy-1-hydroxy-6(E)-tetradecene-8,10,12-triyne.

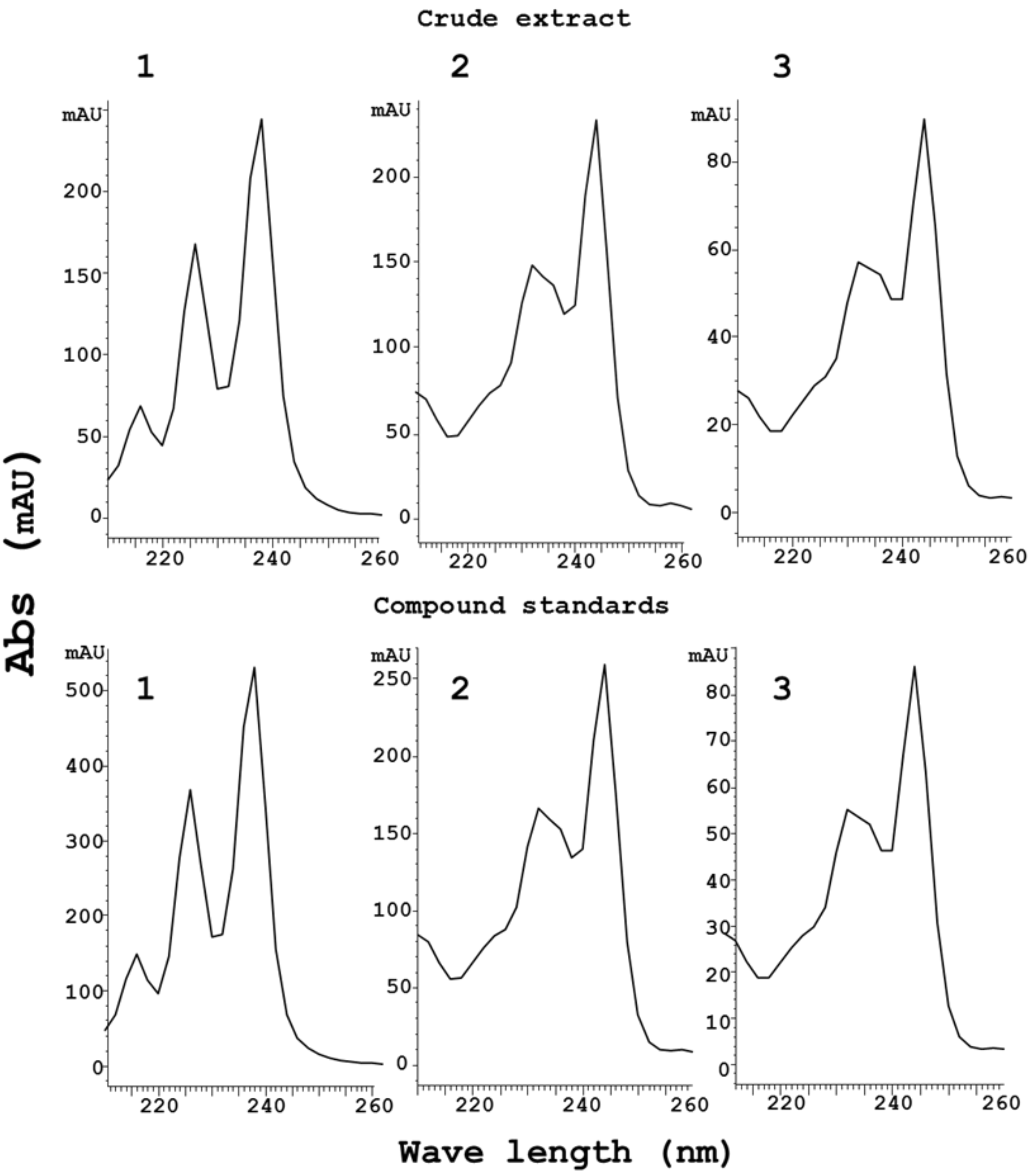

Fig. S4. Ultraviolet spectra of *B. pilosa* extracts and three polyynes. The same extract and compounds were subjected to HPLC analysis, followed by ultraviolet (UV) detection at 210 nm to 260 nm. The UV spectra of peaks 1 to 3 and 3 polyynes are shown. Peaks 1 to 3 correspond to the same peaks in Fig. 1.

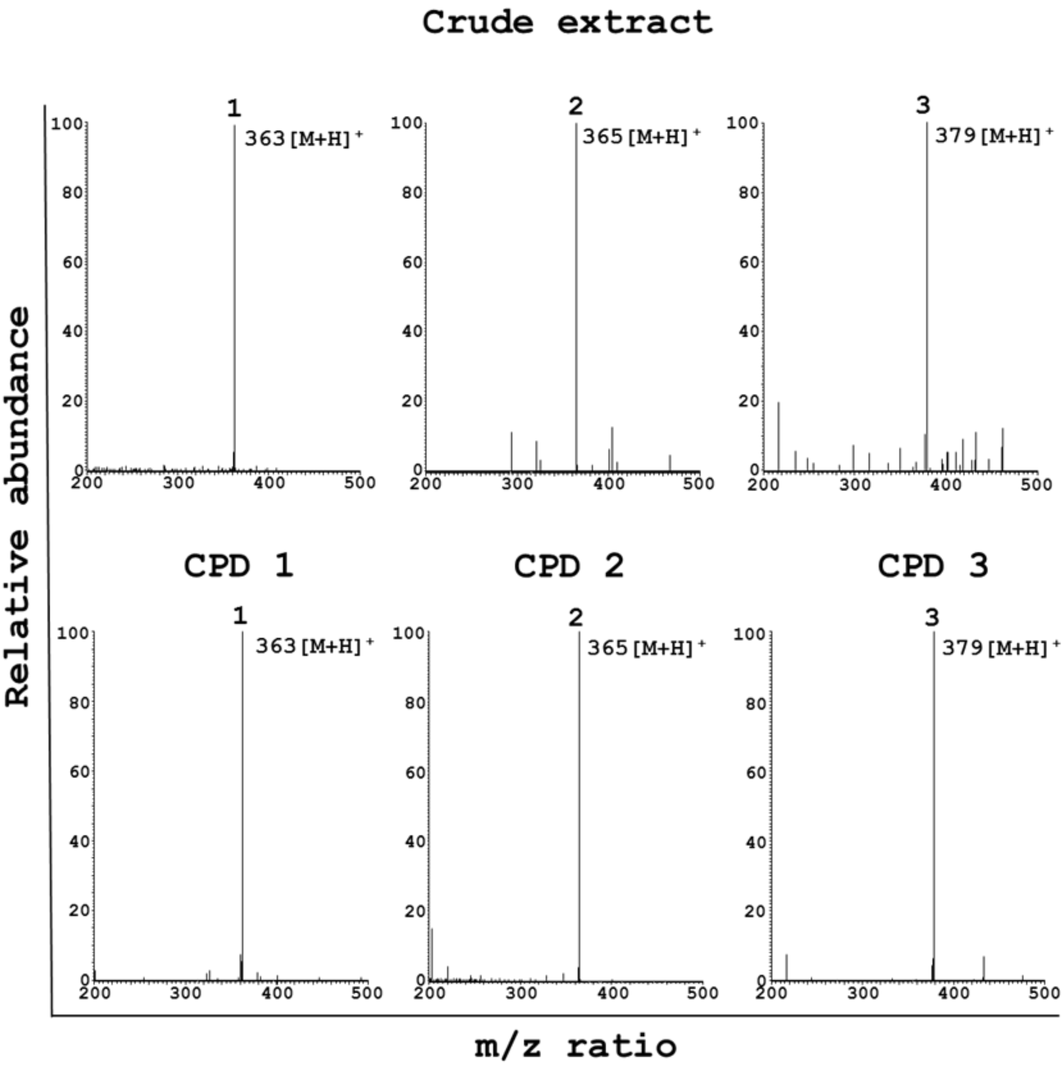

Fig. S5. Mass spectra of *B. pilosa* extracts and three polyynes. The *B. pilosa* extracts and their pure bioactive polyacetylenes were subjected to HPLC-ESI-MS. The Ms scans were performed in positive ion mode (m/z 200 to m/z 500). Peaks 1, 2 and 3 in the crude extracts (top row) and 3 polyynes (bottom row) showed ion signals at m/z 363, 365 and 379, respectively. Peaks 1 to 3 correspond to the same peaks in Fig. 1.

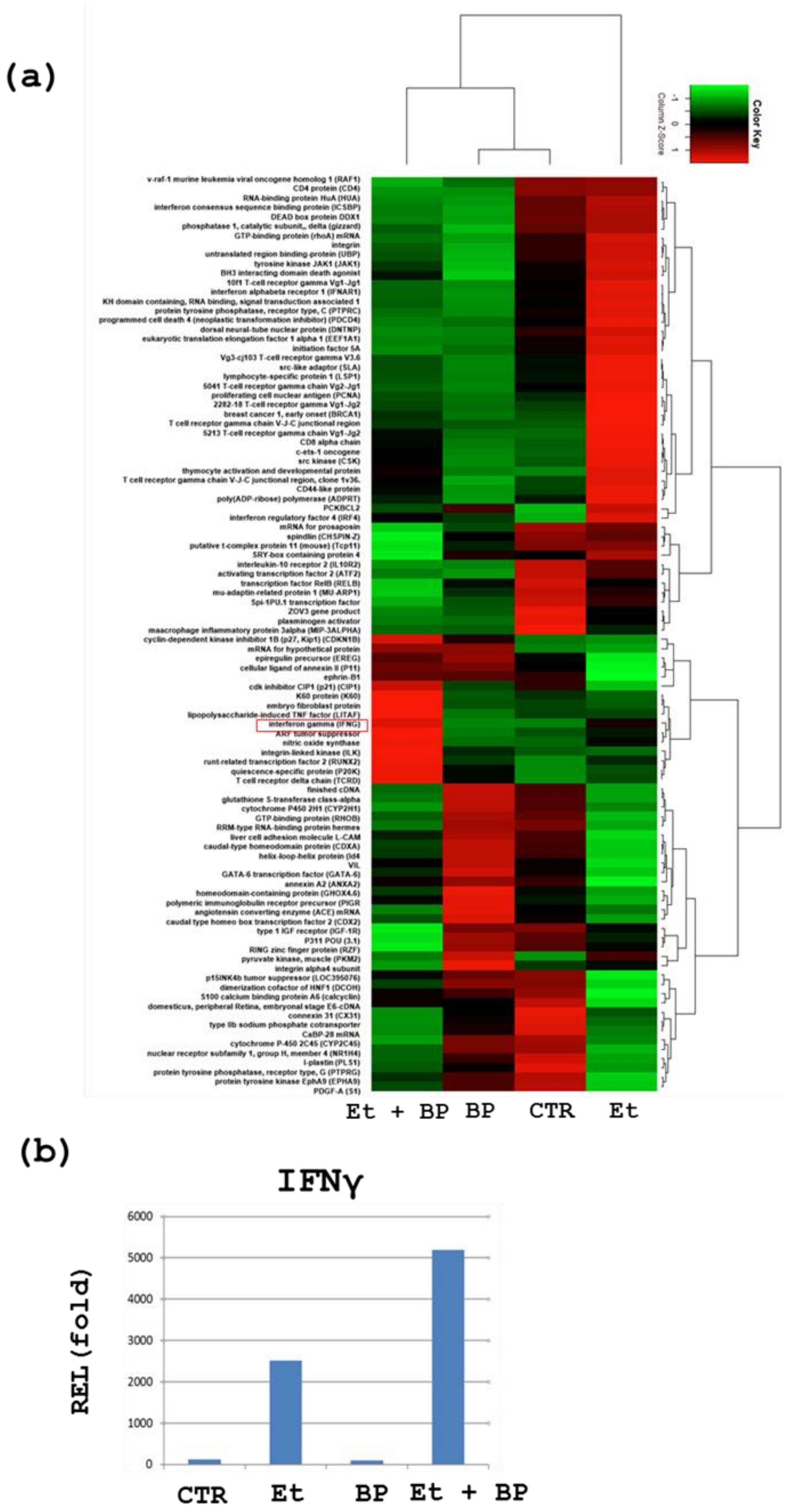

Fig. S6. Impact of *B. pilosa* on T cells using a chicken Affymetrix genechip. T cells isolated from cecal tonsils of chickens fed with standard diet (CTR) and the diet

containing *B. pilosa* diet (0.01%, BP). On day 14, chickens were infected with PBS or *E. tenella* sporulated oocysts (Et or Et + BP) by gavage. The cells from cecal tonsils of 4 groups of chickens were collected for microarray analysis. (a) Cluster analysis and heat map analysis of 100 functionally known genes in T cells from 4 groups of chickens were performed using the R software. (b) Relative expression level (REL) of IFNγ in T cells was quantified.
